# Liangkang Ni on Husserl and Buddhism: a comparative phenomenological analysis

**DOI:** 10.3389/fpsyg.2025.1655534

**Published:** 2025-09-12

**Authors:** Christopher Gutland, Huan Liu

**Affiliations:** Department for Philosophy, Zhejiang University, Hangzhou, China

**Keywords:** phenomenology, Buddhism, Edmund Husserl, Yogācāra, transcendentality, consciousness research, Liangkang Ni

## Abstract

Over several decades, Liangkang Ni has developed a distinctive perspective on the parallels and divergences between Edmund Husserl’s phenomenology and Buddhism, particularly Yogācāra Buddhism. Despite the significance of his contributions, Ni’s writings remain largely unavailable in English and have thus had limited exposure in Western phenomenological discourse. This article addresses that gap by offering a thematic reconstruction of Ni’s key insights. The first part examines Ni’s reading of Husserl’s own philosophical reflections on Buddhism in relation to phenomenology. The second part explores Ni’s reconstruction of genetic and structural parallels between Husserl’s genetic phenomenology and Yogācāra doctrines such as *vijñaptimātra* (consciousness-only), *ālaya-vijñāna*, and *manas*. The article assumes some familiarity with Husserl and uses his framework as an entry point to introduce Buddhist concepts. Rather than offering new empirical data, the article provides conceptual clarification and theoretical integration aimed at informing contemporary debates in consciousness studies.

## 1 Introduction^1^

Over the past three decades, Liangkang Ni has developed a distinctive perspective on the relationship between Husserlian phenomenology and Buddhism, particularly Yogācāra Buddhism. While Ni’s writings are recognized and discussed in Chinese academic circles, they have thus far remained relatively unknown in English-speaking philosophical discourse. One reason is the limited availability of his work in English translation (Ni, 2007, 2010d; Ni, 2010c). This article seeks to address that gap by presenting and reflecting on some of the most thought-provoking aspects of Ni’s approach. The article is divided into two main parts. The first examines Ni’s commentary on Husserl’s own reflections on the affinities and divergences between transcendental phenomenology and Buddhism (Section 2). The second explores Ni’s comparative analysis of Yogācāra Buddhism and Husserlian phenomenology, with a focus on their respective accounts of the structure and transformation of consciousness (Section 3).

Why address this topic in a journal devoted to psychology? Although early psychologists such as Brentano, Külpe, and Binet attempted to develop introspective methods for investigating mental life, psychology has largely abandoned these efforts in favor of behaviorism and verbal report protocols (Depraz et al., 2003, pp. 129–154). In recent years, however, there have been renewed calls to reintegrate rigorous forms of introspection into psychological research (Shear and Varela, 1999; Weger et al., 2019). We argue that the transcendental investigations undertaken by phenomenology and Buddhism are crucial to this endeavor. If psychology is to avoid becoming a “psychology without a soul” (Lange, 1887, p. 465), it must attend to the kinds of methodological guidance that approaches like phenomenology and Buddhism provide.^2^ Husserl saw phenomenology and psychology as intimately related (Husserl, 1976, p. 178), and de Bary writes about Buddhism: “The fundamental truths on which Buddhism is founded are not metaphysical or theological, but rather psychological” (de Bary, 1972, p. 9). Both traditions offer systematic tools for distinguishing—within lived experience—between such phenomena as attention, reflection, awareness, consciousness, dispositions, and acts, distinctions that remain a desideratum in contemporary consciousness research. And even if the deeper strata of consciousness ultimately extend beyond the scope of psychology narrowly conceived, recognizing and articulating this boundary is itself of critical importance for psychological inquiry.

Given the space constraints of this article, we assume that readers are already familiar with the basics of Husserlian phenomenology. Our aim, then, is to build on this familiarity to introduce key Buddhist concepts. This method mirrors the classical Chinese translation practice of *geyi* (格义), which Ni discusses in one of his early articles—a practice in which foreign ideas are initially rendered through familiar categories as a first step toward deeper intercultural understanding.^3^

## 2 Husserl’s own reflections on phenomenology and Buddhism

### 2.1 Husserl and Ni on commonalities between Buddhism and phenomenology

Husserl’s reflection on the relationship between Buddhism and phenomenology was prompted by his review of a German translation of Buddha’s teachings.^4^ Later, in January 1926, Husserl wrote a manuscript titled *Socrates-Buddha* that stands in the context of reflections on “European science vis-à-vis the Indian manner of thinking” (Luft, 2010, p. 4). In it, the name ‘Socrates’ stands for Greek philosophy, and Husserl sees transcendental phenomenology as its continuation (Ni, 2011, pp. 145–146). Believed for some time to be fragmentary (Schuhmann, 2004, p. 151, note 52; Luft, 2010, p. 2), the text was studied only in part (Schuhmann, 2004; Lau, 2010) until Luft discovered the missing pages and published it in full (Husserl, 2010). Ni discusses the full version alongside Husserl’s previous review (Ni, 2011).^5^ In the manuscript, Husserl meanders between describing phenomenology and Buddhism, frequently not indicating the shifts, which is why Ni’s commentary provides a helpful guide through the maze.

Ni highlights that although Husserl lacked expertise, he nonetheless adopted a remarkably “respectful attitude toward Buddhist thought,” paired with a “genuine will to a better understanding of the foreign tradition” (Ni, 2011, p. 143). He argues that *Socrates-Buddha* is noteworthy for offering “Husserl’s transcendental philosophical interpretation of Buddhism” (Ni, 2011, p. 144). Ni also observes that while Husserl’s review was practically oriented—seeking to rejuvenate European spiritual life through engagement with Indian thought—*Socrates–Buddha* presents a more theoretical comparison (Ni, 2011, p. 146).

Ni identifies and evaluates five points of convergence and four differences between phenomenology and Buddhism as articulated by Husserl. We begin by examining the points of convergence that Husserl himself recognized.

#### 2.1.1 Transcendentality

Ni cites Husserl’s description of Buddhism as a form of religious life “purely turned inward in contemplation and striving action—I would like to say, not ‘transcendent’ but ‘transcendental’” (Husserl, 1989, p. 125; Ni, 2011, p. 147). Ni concurs, noting that unlike most other religions, which posit an external, transcendent deity, Buddhism emphasizes “inner insight and practice” (Ni, 2011, p. 147). To support Husserl’s view of a shared transcendental orientation, Ni draws on the *Sandhinirmocana-sūtra*, citing the Buddha’s account of the first two of the ‘six supports’: knowing well the abiding and the arising of thought. He interprets ‘knowing well’ in terms of Husserl’s intuition of essences (*Wesensschau*), while the ‘abiding of thought’ parallels static phenomenology’s investigation of cross-intentionality (*Querintentionalität*). In contrast, the ‘arising of thought’ corresponds to genetic phenomenology’s analysis of longitudinal intentionality (*Längsintentionalität*) (Ni, 2011, p. 147).

#### 2.1.2 Seeing the world as a phenomenon

Another reason why Husserl attributes “transcendentalism” to Buddhism is its view of the world as “merely a phenomenon in subjectivity” (Husserl, 2010, p. 16; Ni, 2011, p. 147). Ni identifies this as a second parallel: the Husserlian *epoché*—the bracketing of worldly existence to focus on the world as phenomenon—mirrors the Buddhist practice of world-renunciation (*Weltentsagung*). As Ni has more recently put it, while the *epoché* constitutes a theoretical call to disinterest in the world, Buddhism advances a practical call to non-attachment (Ni, 2022). He notes that this resemblance is particularly evident in the Yogācāra doctrine of “consciousness-only” (*vijñapti-mātratā*), which is coupled with the affirmation of the “non-existence of external objects” (*anartha*) (Ni, 2011, p. 147). He cites Husserl, who interprets Buddhism as adding a practical dimension to the *epoché*, after which “the ego, withdrawn into itself, lives in willful will-lessness, in theoretical and practical renunciation of the world” (Husserl, 2010, p. 16; Ni, 2011, p. 148). We return to the role of theory and practice in both traditions further down.

#### 2.1.3 Striving for autonomy

Ni quotes Husserl’s remark that, like phenomenology, “Indian cultural life also leads to autonomy—to autonomous knowledge, through which an inherently true path to bliss, through which truth in itself for right action, autonomous truth in the knowledge of ethical-religious norms, can be gained.” (Husserl, 2010, p. 5; Ni, 2011, p. 148) Ni concurs, emphasizing that in Buddhism, the distinction between philosophy and religion is absent. He adds: “Everyone is their own inner judge and is not governed by external laws. In this sense, it becomes clear once again that Buddhism is not transcendent, but transcendental, that is, ‘purely inward-looking’” (Ni, 2011, p. 149).

#### 2.1.4 Universal truths

Ni next turns to Husserl’s observation that both Buddhism and phenomenology “intend a kind of general reflection or a transcendental generality” (Ni, 2011, p. 149). Yet here, a first difference emerges: phenomenology is epistemically oriented, whereas Buddhism is ethically oriented. Ni explains: “The epistemic attitude proves to be a general ‘theoretical interest’ directed toward the truth of judgment and having its correlate in being-in-itself; correspondingly, the ethical attitude proves to be a general ‘ethical interest’ directed toward the truth of the will and having its correlate in goodness-in-itself.” (Ni, 2011, p. 149) We discuss this difference in more detail in Section 2.2.

#### 2.1.5 Intuition of essences

Ni highlights how Husserl praises “Buddha especially for ‘his consistency, his lack of prejudice, his determination […] in expressing his evaluations in judgments of essence’” (Husserl, 2010, p. 13; Ni, 2011, p. 149). Although Husserl refers only to judgments of essence, Ni assumes Husserl attributes to Buddhism also the capacity for the intuition of eidetic laws.^6^ Buddhism’s practical orientation, however, again marks a point of divergence. As Husserl notes, “for such an Indian attitude there is no world science as a goal, and knowledge of truth has meaning only as knowledge directed toward establishing the transcendental standpoint, that is, the world as a phenomenon, furthermore toward the most general nature of universal will-life in general and toward its possible purpose.” (Husserl, 2010, p. 16; Ni, 2011, p. 150).

The attentive reader will have noticed a tension in Husserl’s characterization: How can a Buddhist be ‘withdrawn into themselves, live in willful will-lessness, in theoretical and practical renunciation of the world,’ while simultaneously being directed toward the ‘truth of the will’ and ‘goodness-in-itself’? This tension stems from Husserl’s distinction between rationality and irrationality, which leads us to the four key differences between Buddhism and phenomenology that Ni identifies in Husserl’s perspective.

### 2.2 Ni on the distinctions between Buddhism and phenomenology as seen by Husserl

#### 2.2.1 Formalization or logification

Despite acknowledging Buddhism’s transcendental orientation and eidetic insight, Husserl writes that “for the Indians, the thinking of the doctrine of salvation was not distinguished in its form (and logic, so to speak) from natural thinking” (Husserl, 2010, p. 5; Ni, 2011, p. 151). Ni therefore notes that a “key criticism of Husserl’s Buddhist doctrine of salvation is that it has not undergone any formalization or logification, as is known from ancient Greek philosophy” (Ni, 2011, p. 150). He rightly counters Husserl’s assessment by pointing out that later Buddhist traditions developed sophisticated logical systems, such as *Hetuvidyā* and *Nyāya*. Husserl likely raised this point because the Buddhist texts he knew lacked treatises on logic.

#### 2.2.2 The foundational relation between theory and practice

Husserl holds that theory—understood as *First Philosophy*—is the foundation of practice. Viewing the Buddhist as existing “in a universal practical attitude” (Husserl, 2010, p. 17; Ni, 2011, p. 152), he “considers phenomenology to be a general epistemology and Buddhism to be a general ethics” (Ni, 2011, p. 152). If this is correct, then “phenomenological philosophy and Buddhist thought” would stand “in the relationship between foundational and founded” (Ni, 2011, p. 152).

This claim warrants deeper reflection. We wish to expand and support Ni’s discussion with some additional considerations. Ni refers to the *Crisis*, where Husserl equates practically oriented sciences with a mere *techné* (τέχνη), lacking the striving for universality (Husserl, 1962, p. 201; Ni, 2011, p. 152). A similar stance appears in *Socrates–Buddha*, where Husserl writes: “Practice is limiting—in general. […] Trying to solve epistemological problems with a finite, practical purpose never results in science” (Husserl, 2010, p. 9). Here, Husserl positions phenomenology in opposition to a pragmatic attitude. After all, the *ends-in-view* or foreseen consequences of my practical attempt to solve a given problem may be fully realized, even if the epistemic assumptions underlying my conduct are flawed. This is because in the pragmatist framework, the adequacy of a perspective is measured not by being true to ‘*the things themselves*’, but by its operational success. For instance, as previous centuries tell us, one can *practically* navigate much of one’s everyday life effectively while believing the Earth to be flat. This exemplifies what Husserl finds lacking in pragmatic views: they bypass the question of whether the epistemic intentional comportment toward the world is faithful to the things themselves. Conversely, pragmatism renders Husserl’s epistemological standard superfluous by prioritizing practical success in the external world over truth.

Buddhism, however, is not about solving *worldly* problems. Its transcendental orientation sets its practical goals on a plane fundamentally different from that of pragmatism. This distinction prepares the ground for Ni’s critique of the foundational relation between theory and practice that Husserl advocates. Ni argues “that scientific endeavors also represent a kind of practice” (Ni, 2011, p. 153). Somewhat surprisingly, however, he does not cite the sentence in *Socrates–Buddha* that most directly supports his point. Husserl writes: “Every epistemic truth corresponds to a practical truth, if it is true that every judgment directed toward truth is, as a practical action, a practical truth” (Husserl, 2010, p. 15). This suggests that the act of forming true judgments is itself a kind of practice—albeit a mental one. Unlike common forms of practice aimed at changing the external world, this epistemic practice involves an *inward transformation*. In seeking truth, the subject changes not the external world, but itself: it needs to originally institute (*urstiften*) within itself new judgments, concepts, or types such that *its conscious experience develops* toward better aligning with the world *as it is*.

On the one hand, Husserl is right to insist that the ideal basis for effective external action is accurate knowledge of external states of affairs. The less we understand the situation we face, the less likely we are to achieve our aims. In this sense, theory provides the foundation for practice. But in another sense, the act of acquiring new apperceptive types and concepts to then form true judgments is itself a kind of inner practice that grounds epistemic knowledge. We believe that this clarification of the dual meanings of ‘practice’ we added here deepens the understanding of both Ni’s and Husserl’s views, while also resolving the tension in Husserl’s account. Specifically, Husserl sees Buddhism as ‘willful’ in its inward pursuit of ethical autonomy and truth, but ‘willless’ in terms of external conduct.

#### 2.2.3 Striving for theoretical versus practical autonomy

Since Husserl identifies a difference in how both traditions value theory and practice, he also discerns a corresponding difference in the form of autonomy they pursue. He sees phenomenology as striving for “epistemic autonomy,” while Buddhism aims at “practical autonomy” (Ni, 2011, p. 153; Husserl, 2010, p. 6; Ni, 2009, pp. 37–38). Epistemic autonomy consists in forming judgments based on evident self-givenness, rather than accepting claims based on external authority (Ni, 2011, p. 154). By contrast, as Ni summarizes Husserl’s view, the “autonomy sought by the Buddha is based on insight into a practical truth” (Ni, 2011, p. 154).

To further question Husserl’s assumption that practice is founded in theory, Ni turns to Husserl’s statement that a “theoretical interest can also be called ‘autonomous’ insofar as the subject regards a consistent search for truth in the sense of finality as an absolutely practical value” (Husserl, 2010, p. 13; Ni, 2011, p. 155). This connects to the earlier discussion: the practical value Husserl assigns to truth is not relative to problem-solving utility, as in pragmatism, but expresses an inwardly directed epistemological commitment—truth-seeking as a practical endeavor carried out in transcendental constitution. As Ni pointedly remarks, “interest in a disinterested observation is also a form of interest” (Ni, 2011, p. 155).

Here, Ni offers a particularly intriguing argument: Since this inner ‘practice’ concerns the constitution of knowledge structures, it properly belongs to genetic phenomenology (Ni, 2011, p. 156). By contrast, Husserl’s usual assumption—that theory grounds practice—is consistent within the framework of static phenomenology, where preexisting structures of knowledge are taken for granted (Ni, 2011, p. 155).

#### 2.2.4 Rationalism versus irrationalism

Ni acknowledges that the final difference identified by Husserl is the most profound. He cites Husserl’s observation: “In contrast to the ‘rationalism’ of Greek science and an ethics that bases philosophical life on philosophical knowledge […], the Indian’s gaze rests precisely on the irrational” (Husserl, 2010, p. 16; Ni, 2011, p. 156). Ni first clarifies that Husserl does not employ the term ‘irrational’ pejoratively (Ni, 2011, p. 156). On the contrary, he highlights Husserl’s account of Buddhism’s contemplative “willful will-lessness,” which makes “all contradictions between rationality and irrationality disappear” (Husserl, 2010, p. 16; Ni, 2011, p. 158). Based on this, Ni suggests that Buddhism transcends the rational-irrational dichotomy, approaching a kind of “trans-rationalism” (*Überrationalismus*) (Ni, 2011, p. 158; Ni, 2009).

### 2.3 Summary and transition to Ni’s own view

Ni succinctly summarizes the core divergence between the two traditions as follows: “There is an immanent relationship between the two central Buddhist concepts of ‘enlightenment’ (*bodhi*) and ‘emptiness’” (*śūnyatā*) such that “their connection signifies insight into absolute non-being. In contrast, in phenomenology, there is an immanent relationship between the two central concepts of ‘evidence’ and ‘pure consciousness,’ which express insight into absolute being” (Ni, 2011, p. 158).

We would like to add: Although the *epoché* brackets the world, Ni himself emphasizes that it does not negate it (Ni, 2010a, p. 240). Its function is epistemic rather than ascetic or practical. Consequently, ethical conduct *in the external world* remains a positive ideal for Husserl. To engage in such conduct, one must step out of the *epoché*, as moral action in Husserl’s sense requires a practical involvement with worldly existence. From this perspective, the Buddhist practical aspiration to gain insight into absolute non-being, when contrasted with the ethical imperative to realize the good in the external world, may appear—at least from a Husserlian standpoint—as ‘irrational.’ However, Buddhism, too, has a robust ethical framework, grounded not in worldly teleology but in the alleviation of suffering. As de Bary notes, “the question of ‘helping’ or ‘saving’ others is presented specifically as a question of teaching, i.e., of sharing Enlightenment” (de Bary, 1972, p. 69). Accordingly, ethical action in Buddhism takes the form of compassionate guidance. Yet our aim is not to evaluate these ethical frameworks against one another, but to underscore how their respective philosophical premises give rise to differing conceptions of ethical life.

The foregoing summary of Husserl’s account of the relation between Buddhism and phenomenology—together with Ni’s assessment—offers a helpful point of departure for turning to Ni’s own position. In contrast to Husserl, Ni focuses primarily on Yogācāra Buddhism. Since he elaborates his view with varying degrees of detail across different publications, the following section combines insights from several of his articles.

## 3 Ni on the relationship between phenomenology and Yogācāra Buddhism

The previous section showed that Husserl himself saw affinities between Buddhism and phenomenology, even classifying Buddhism as a form of transcendentalism due to its orientation toward consciousness and its treatment of the world as a mere phenomenon. Building on this foundation, we can now outline how Ni conceives of the relation between the two traditions. To avoid repetition, we will not restate similarities and differences already noted. Let us start with a brief introduction to Yogācāra Buddhism.

### 3.1 Introducing Yogācāra Buddhism and some preliminary reflections

Yogācāra Buddhism emerged in India around the 4th and 5th centuries CE and was systematized by the half-brothers Asaṅga and Vasubandhu. Ni describes it as “the earliest systematic philosophy of consciousness” (Ni, 2010a, p. 221).

One of its central doctrines is *vijñaptimātra*—the idea that there is only consciousness. While this may initially appear more radical than phenomenology, Zahavi notes that some interpreters, drawing on Husserl’s claim that “the world is conditioned by transcendental subjectivity,” regard Husserl’s transcendental idealism as “an internalism so radical that it undermines the traditional gap between mind and world, thereby approaching a form of externalism” (Zahavi, 2004, p. 51). Though certainly not the only interpretation of Husserl, this shows the point of a potential convergence between the two views. Along similar lines, de Bary cites a key Mahāyāna tenet from the *Ratnamegha Sūtra* that echoes Husserl’s epistemological orientation: “All phenomena originate in the mind, and when the mind is fully known all phenomena are fully known” (de Bary, 1972, p. 100).

The view that there is only consciousness stands in stark contrast to the materialist assumptions intersubjectively embodied in the prevailing scientific horizon. These assumptions typically hold that matter must exist first and that consciousness either emerges from it, is reducible to it, or constitutes a mere epiphenomenon. Yogācāra, by contrast, denies the existence of external objects altogether (*anartha*), thereby rejecting the very basis for materialism.^7^ Moreover, even within consciousness, Yogācāra reverses assumptions common in Western philosophy: Ni points, for instance, to Locke, who held that reflection presupposes sensation—whereas in Yogācāra, reflective consciousness is not built on sensory input but arises in a different, more originary manner (Ni, 2010b, pp. 253–254).

Yogācāra posits a genetic unfolding of consciousness in three stages (*trividha-pariṇāma-samutpāda*) comprising eight types of consciousness. Ni explains that the defining characteristic of all kinds of consciousness is “the ability to distinguish or identify” (Ni, 2010b, p. 246). While Ni usually follows the genetic order, we begin here with everyday consciousness as phenomenology examines it, before turning to the deeper layers assumed in Yogācāra.

### 3.2 The Sixfold ordinary consciousness (*ṣaḍvijñāna*)

Ni numbers the types of consciousness inversely to their genesis. The first five—sight, touch, taste, smell, and hearing—are the last to emerge developmentally. The sixth consciousness is “intellectual consciousness, which can also be described as consciousness of reason or understanding” (Ni, 2010a, p. 234). It synthesizes the five senses and is called *mano-vijñāna*. Ni explains: “The relationship between the first five types of consciousness and spirit-consciousness is similar to that between pure sensations and apprehensive perception. […] Only in perception, i.e., accompanied by spirit-consciousness, does a unified object emerge from sensations” (Ni, 2010b, p. 248).

This parallels Husserl’s distinction between *Empfindungen* (sensations) and *Auffassung* (apprehension), where only the latter constitutes consciousness of objects in the sense of things, often by apprehending data from multiple sense fields. Yet, whereas “Husserl would rather describe the first five types of consciousness as hyle or sense data,” Yogācāra regards them as “acts of consciousness” (Ni, 2010a, p. 234). Ni concurs with Husserl, seeing the first five consciousnesses not as acts, but as mere sense data that depend on the sixth consciousness and co-occur with it (Ni, 2020, p. 92).

According to Xuan Zang (玄奘, 602–664 CE), the five senses distinguish sensory materials, while the sixth consciousness “distinguishes all laws and rules” (Ni, 2010b, p. 247). The defining feature of everyday consciousness is object-positing, which is “the reason why some Yogācāra Buddhists define the doctrine of the six types of consciousness as epistemology” (Ni, 2010a, p. 234). Husserl’s notion that “every consciousness is ‘consciousness of’” thus applies well to this stage (Husserl, 1989, p. 16; Ni, 2010a, p. 234).

Ni finds further parallels in structural analysis. Both Husserl and Yogācāra, he argues, acknowledge a threefold structure in experience: noesis, noema, and self-consciousness (Ni, 2010a, p. 238).^8^ Self-consciousness (*svasamvitti-bhāga*) denotes “the non-objective awareness of the act itself in its execution” (Ni, 2010b, p. 251), akin to inner consciousness in Husserl (1966, pp. 126–130) and Brentano (2008, pp. 118–119).^9^

Another parallel is the foundational relation within the six consciousnesses. Husserl, following Brentano, holds that “every act is either a presentation or has presentations as its basis” (Husserl, 1984, p. 354; Brentano, 2008, pp. 97, 103; Ni, 2010d, p. 238). Yogācāra frames this in terms of *citta* (objectifying acts) and *caitta* (non-objectifying acts that presuppose objects). Ni explains: “Non-objectifying acts can only come about through objects constituted by objectifying acts. For example, enjoyment without something to be enjoyed is unthinkable” (Ni, 2010a, p. 225). Ni thus argues the pair *citta–caitta* “corresponds exactly to Husserl’s understanding of the static, cross-wise foundational relationship in the *Logical Investigations*” (Ni, 2010a, p. 238; Ni, 2015, p. 57).

While Husserl did not catalog these dependencies, Yogācāra recognizes “six classes and fifty-one types” of *caitta* (Ni, 2010a, pp. 238, 224). However, Ni notes that Husserl explored “the gradual foundational relationship between perception, imagination, image consciousness, and sign consciousness” (Ni, 2010a, p. 239)—a structure Yogācāra does not articulate. This is one of several reasons Ni advocates for dialogue between the two traditions.^10^

Ni sums up: “The conscious experiences with which Husserl deals in his published phenomenological analyses are primarily those that belong to the six types of consciousness in Yogācāra Buddhism, i.e., to object-consciousness” (Ni, 2010a, p. 235). But Yogācāra posits further, deeper layers of consciousness. This reflects its Mahāyāna background, in contrast to Hīnayāna Buddhism, which recognizes only the six consciousnesses (Ni, 2010a, pp. 226–227). But why assume anything beyond everyday consciousness?

### 3.3 *Manas*—the seventh consciousness

The seventh consciousness is referred to as *manas* or *manas-vijñāna*. While the latter term resembles that of the sixth consciousness—*mano-vijñāna*—unlike the sixth (which corresponds to reason or understanding), the seventh is not analogous to Descartes’ *cogito* or Husserl’s noesis (Ni, 2010a, p. 230). Rather, it accounts for the continuity of consciousness across states devoid of reasoning. Upon waking from dreamless sleep, we still presume ourselves to be the same person. Yet during such sleep, there is no discursive thought. If our existence depended on Descartes’ *ego cogito*, we would effectively cease to exist each night.

Ni explains that *manas* enables “continuity and identity of consciousness”—it is why “after sleep, passing out, etc., the awakened subject of consciousness will still consider itself the same ego” (Ni, 2010a, p. 231). Ni equates *manas* with Husserl’s pre-ego (*Vor-Ich*), “the non-objective ego-consciousness before reflection” (Ni, 2010a, p. 231). He further sees it as the *subiectum* underlying “skills you have without practicing, knowledge you have without learning” (Ni, 2010a, p. 242). Drawing on Taguchi (2006), Ni also portrays *manas* as a “phenomenology of nature” distinct from a “phenomenology of nurture” (Ni, 2010a, p. 232). However, Ni rejects a strict nature–nurture split, noting that habitualization allows nurture to sediment into nature, a reason why some Yogācāra Buddhists consider the related research as anthropology (Ni, 2010a, p. 233).

The sixth consciousness (*mano-vijñāna*) unifies the five senses and can, over time, transform from nurture into nature. It thus serves as a link between the five sense-consciousnesses and the seventh consciousness (*manas*) (Ni, 2010b, p. 248, note 2). Ni contrasts the sixth and seventh consciousness by observing that the sixth “can be characterized as non-continuous and non-egoic, while the reverse is true for the seventh” (Ni, 2010b, p. 249).

### 3.4 *Ālaya*—the eighth consciousness

Why posit an even deeper, more primordial consciousness? Is our existence not adequately captured by the transition between sleep and wakefulness?—Not according to Yogācāra Buddhism, which affirms the cycle of rebirth (*saṃsāra*), grounded in “the identity of consciousness and its continuity” even across successive incarnations in “different bodies” (Ni, 2010a, p. 230, Ni, 2010d, p. 270). While the notion of reincarnation may seem alien to much of Western philosophy, the idea of an immortal soul is not. Brentano, for instance, considered the capacity to take a well-founded stance on the question of immortality to be among the fundamental requirements of psychology (Brentano, 2008, pp. 30–35).

Yet does phenomenology provide the resources to address this issue? In one text, Ni writes that the “soul in its cycle belongs in principle to problems that phenomenology has to exclude” (Ni, 2010a, p. 236). Elsewhere, however, Ni cites a manuscript in which Husserl himself moves from sleep to death in his line of thinking, asking: “[D]oes death not remain the brother of sleep? […] Man necessarily dies. But the transcendental, primal life, the ultimately world-creating life and its ultimate ego, cannot arise from nothing and pass into nothingness; it is immortal, because dying has no meaning for it” (Husserl, 1993, p. 338; Ni, 2010d, p. 268). In *Ideas I*, Husserl asserts that the destruction of the natural world would modify consciousness, but not abolish its existence (Husserl, 1976, p. 104). In 1930, he even cautiously contemplated reincarnation (*Seelenwanderung*) (Husserl, 2008, pp. 224–230). For Husserl, then, consciousness belongs to a plane of existence distinct from that of physical nature—perhaps explaining his surprising receptivity to certain Buddhist teachings (Husserl, 1989, pp. 125–126).

Yet, how to conceive of the eighth consciousness—*ālaya*—in Husserlian terms? In the quote above, Husserl referred to the ‘ultimate ego’ (*letztes Ich*). Accordingly, Ni equates *ālaya-vijñāna* with Husserl’s talk of the ‘primal ego’ (*Ur-Ich*)^11^ and the ‘ultimate consciousness’ (*letztes Bewusstsein*) (Ni, 2010a, pp. 222, 227; Ni, 2010d, p. 265; Ni, 2018b, p. 54). Ni further associates it with the living present (*lebendige Gegenwart*), a pre-reflective, pretemporal mode of being. Quoting Held, he equates it to “the mode of being of the ultimately functioning transcendental ego (primal ego)” that constitutes without being constituted (Held, 1974, p. 138; Held, 1966, p. 63; Ni, 2010a, p. 229). Because *ālaya* is regarded as the most fundamental stratum, some interpret related investigations as ontological in nature (Ni, 2010d, pp. 222–223, 226).

Ni distinguishes the non-objectifying acts associated with *ālaya* and *manas* from those of *caitta*, proposing that they occur either before or after the objectification process within the sixth consciousness. Accordingly, *ālaya* and *manas* function as pre-objectifying acts, while *caitta* represents post-objectifying acts (Ni, 2010a, p. 242). Consistent with this, he also suggests that *ālaya* and *manas* underlie the genesis of pre-reflective meaning sedimentation in objectifying acts (Ni, 2020, p. 94).

Ni also underscores important differences. In Yogācāra, *ālaya* ensures unity and continuity throughout the cycle of rebirth (*saṃsāra*), thereby grounding a persistent monadic identity. By contrast, Husserl’s primal ego is intermonadic (Ni, 2010a, p. 230, note 1). Furthermore, Yogācāra conceives *ālaya* and *manas* as genetic strata that precede ordinary consciousness. Yet these layers do not simply disappear once everyday object-consciousness arises; rather, they remain operative as structural components within it. As Ni observes, Husserl, insofar as he acknowledges analogues to *manas* and *ālaya*, does not treat them as distinct genetic stages, but as functional structures immanent to the sixfold object-consciousness (Ni, 2010a, p. 235). This divergence reflects, at least in part, differing methodological commitments in Yogācāra and phenomenology—a contrast to which we now briefly turn.

### 3.5 Differences in methodology

Ni goes so far as to claim that “the Yogācāra School discusses *Ālaya-vijñāna* thoroughly and completely” (Ni, 2010d, p. 270). By contrast, Husserl remains cautious about the primal ego, constrained by his “principle of all principles”—the imperative to base all claims on direct intuitive givenness (Husserl, 1976, p. 51; Ni, 2010a, p. 229; Ni, 2010d, p. 266). Already in his early work, Husserl warned against invoking “the ever-convenient unconscious” as an explanatory device (Husserl, 1970, p. 254).

Yogācāra, by contrast, when articulating the nature of *ālaya*, typically “appeals to deduction in addition to classical texts” (Ni, 2010d, p. 266). From a Husserlian perspective, such constructs risk becoming mere ‘logical substructions’ (Husserl, 1962, p. 130). Nevertheless, Ni rightly observes that Husserl himself engaged in deductive reasoning, especially in his genetic phenomenology (Ni, 2010d, pp. 273–274). And yet, Ni argues that the “‘perceiving’ of *Ālaya-vijñāna* is […] theoretically possible in Yogācāra Buddhism” (Ni, 2010d, p. 274).

This divergence presents a fundamental choice: Should we posit non-experiential structures of consciousness through speculative deduction, or should we instead strive to expand and refine our actual experience of consciousness? We advocate for the latter—on two main grounds. First, the principle of all principles is phenomenology’s core strength, distinguishing it from approaches like Freud’s psychoanalysis. Rather than expanding experience, psychoanalysis maintains the boundary between conscious and unconscious, populating the unconscious with speculative constructs that, in effect, are modifications of the known. While psychoanalysis may achieve therapeutic success within pragmatic standards, it does so by interpreting the unconscious in terms of the familiar, thus creating the illusion of uncovering something new. In contrast, a phenomenology that stays true to actual experience seeks to uncover strata of consciousness that typically escape notice, willing to extend and refine its knowledge based on what it reveals. This is where Buddhism offers significant inspiration for expanding experience. Conversely, if phenomenology adopted a hypothetico-deductive framework, it would succumb to the Duhem–Quine problem of underdetermination, which plagues empirical sciences due to the arbitrariness of hypothesis formation (Carrier, 2009, pp. 20–21). In Husserl’s words, such theorizing risks overlaying the world with an “ideal garment” that obscures rather than reveals the things themselves (Husserl, 1962, p. 51).

While there may be a limit to how far conscious experience can be deepened, we have certainly not reached it. We suggest that the promise of combining phenomenology and Buddhism lies in the potential to broaden and deepen our access to experiential structures of consciousness. To illustrate: Wenjing Cai has persuasively argued that Husserl, over time, came to privilege apodicticity over adequacy (Cai, 2013). Nevertheless, Husserl still speaks of an “*insight* of apodictic necessity” (Husserl, 1956, p. 14, emphasis added), implying that the notion of ‘intuition’ can and must be broadened beyond mere sensibility (*Sinnlichkeit*).^12^

The second reason is simple: If *ālaya* is construed either as a dogmatic postulate or as a purely deductive construct—in either case lying in principle beyond experiential access—then any system that relies on it ceases to be transcen*dental* and becomes transcen*dent*. In contrast, we advocate a more charitable reading of Buddhist texts, one that presumes their authors aimed to articulate genuine experiential insights rather than formulate speculative metaphysics. On this view, such texts are not to be accepted blindly, but engaged with as practical guides—maps pointing toward structures that may become accessible within one’s own consciousness.

## 4 Summary and outlook

Liangkang Ni consistently emphasizes the striking structural parallels between Husserlian phenomenology and Yogācāra Buddhism (Ni, 2010a, p. 242; Ni, 2010d, p. 261; Ni, 2010b, p. 257). This culminates in his bold suggestion that phenomenology might better be described as a “Yogācāra Buddhism of the twentieth century” rather than a “new Cartesianism” (Ni, 2010a, p. 243). As we have seen, Husserl himself recognized remarkable affinities with Buddhist thought, while also highlighting crucial differences.

Building on these insights, we have offered a thematic reconstruction of Ni’s interpretation of the relationship between Husserlian phenomenology and Buddhism, particularly Yogācāra Buddhism, highlighting both structural parallels and philosophical divergences. We argued that Ni’s reading reveals convergences in their transcendental orientation and models of consciousness, while also uncovering tensions concerning rationality, the relation between theory and practice, and methodological commitments. Drawing on this comparison, we proposed several conceptual clarifications that extend the dialogue between these traditions and point toward a more experience-based integration of introspective methodologies in consciousness studies.

These insights, while grounded in philosophical analysis, also carry relevance for adjacent fields such as psychology. By highlighting structural and methodological parallels between phenomenology and Buddhist introspective traditions, Ni’s work encourages a reconsideration of first-person methods as legitimate tools for investigating mental life. This perspective supports ongoing efforts to reintegrate disciplined forms of inner observation into psychological research—an endeavor that could enrich not only theoretical models of consciousness but also practical approaches to mental well-being.

Given the complexity of comparing traditions rooted in distinct cultural and conceptual horizons, no single interpretation can claim final authority. As phenomenology teaches, genuine understanding requires viewing a phenomenon from multiple sides. Similarly, grasping the relation between phenomenology and Buddhism benefits from engaging a variety of perspectives. In addition to Ni, scholars such as Coseru (2025), Kern (1988), Larrabee (1981), Lau (2016b), Li (2022), Lusthaus (2002), Nishida (1987, 1990), and Sharf (2016) offer complementary insights that enrich this comparative endeavor.

Our focus here has necessarily been selective, setting aside many of the more nuanced developments in Ni’s evolving work. Nevertheless, we hope to have conveyed something of its philosophical depth and contemporary relevance—enough, perhaps, to invite further reflection within the ongoing dialogue between phenomenology and Buddhist thought.

## Footnotes

^1^Unless otherwise indicated, all translations from non-English sources are our own.

^2^Ni identifies introspection as a shared feature of both traditions (Ni, 2010d, p. 259). While some seek to dissociate phenomenology from introspection, we agree with Ni for reasons discussed in Gutland (2018).

^3^In *geyi* (格义), familiar expressions from indigenous traditions are used to render foreign concepts intelligible. For instance, the Daoist notion of *wu* (无, nothingness) was used to translate the Buddhist term *śūnyatā* (emptiness). Ni analyzes this method through Husserl’s concept of *appresentation*: just as the consciousness of the other is not directly presented but appresented (Husserl, 1973, pp. 137–149), foreign concepts are initially understood via similarity associations with familiar ones. While the analogous concept is presented, the unfamiliar meaning is only appresented—left open for further fulfillment. Ni sees this as a necessary first step in intercultural understanding, while also acknowledging the risk of superficial or distorted assimilation. He warns, citing Holenstein (1985), p. 121, that such practices can subordinate foreign meanings to domestic categories. Yet, drawing on Klaus Held, Ni also emphasizes that if pursued with openness, this may lead to a transformation that, phenomenologically speaking, can shift the boundary between self and other, between the familiar and the foreign (Ni, 1998a).

^4^Husserl’s review was first published in 1925 in the *Piperbote* and later reprinted in Husserl (1989), pp. 125–126.

^5^Lau has since revised his article to likewise reflect the manuscript in its entirety (Lau, 2016a).

^6^This is likely because Ni concurs with Zongsan Mou’s (牟宗三, 1909–1995) assessment that intellectual intuition is underlying not only all Chinese philosophy, but also Buddhism (Ni, 2010c, p. 204). Note that this article’s English is currently in a poor editorial state – we recommend interested readers to wait until the second edition of Ni’s book *Zur Sache des Bewusstseins* is published, likely in 2026.

^7^Notably, Indian thought includes systems that posit a substance distinct from consciousness. For instance, Sāṃkhya philosophy—a dualist system often contrasted with the idealism of Yogācāra—holds that primordial nature (*prakṛti*) undergoes a structured evolution, descending from subtle, intellectual principles to gross elements (*mahābhūtas*) (Larson, 2011). Thus, even in frameworks that assume a mind-external substance, physical matter as we ordinarily conceive it emerges only at a very late stage.

^8^Ni’s terminological identifications vary. He equates noesis and noema with *dṛṣṭi-bhāga* (the seeing) and *lakṣaṇa-bhāga* (the seen) (Ni, 2010a, p. 224), with *grāhaka* (the grasping) and *grāhya* (the grasped) (Ni, 2010d, p. 260), or with *darśanabhāga* (the act of seeing) and *nimittabhāga* (the appearing phenomenon) (Ni, 2010b, p. 250).

^9^Chinese Yogācāra Buddhism typically posits a fourth element—consciousness of self-consciousness—to avoid an infinite regress (Ni, 2010b, pp. 250–253, Ni, 2010d, p. 271, Ni, 2010d, p. 224, Ni, 2018b, p. 49). However, Ni suggests that this element is likely superfluous (Ni, 2010b, p. 252, note 1, Ni, 2010a, p. 224, note 2), referring to his own research on reflection (Ni, 1998b). One might argue that Brentano already successfully avoided the problem of infinite regress in self-awareness without introducing such an additional element (Brentano, 2008, pp. 139–148).

^10^Ni’s own later research provides an example for such dialogue. Inspired by the Yogācāra doctrine of *citta* and *caitta*, Ni uncovers a nuanced phenomenological relationship between perception, value-sensing, and willing. He finds that, contrary to his previous view, *caitta* in Yogācāra can also constitute their own objects (Ni, 2018a, pp. 119–120). Acts such as value-sensing and volition, classified as *caitta*, are thus not non-objectifying: each is directed toward a correlate, such as the valuable or the to-be-done. Moreover, because *caitta* always accompanies *citta*, there is no clear foundational asymmetry between them. This insight prompts Ni to identify a parallel in Husserl’s later genetic phenomenology. Unlike the *Logical Investigations* or early static analyses, Husserl here holds that there is ultimately a single act of consciousness that unfolds in different modalities—perceiving, valuing, willing. When one mode dominates, the entire act takes on its character accordingly, and the object constituted through it is likewise modified in its salience and structure (Ni, 2018a, pp. 122–124).

^11^While Yogācāra typically presents *ālaya* and *manas* as genetically linked stages, Ni clarifies that Husserl makes no such genetic connection between the pre-ego and the primal ego (Ni, 2010a, p. 230, note 2, 241).

^12^For an attempt to phenomenologically distinguish the somewhat arbitrary psychological act of judging from intuiting essences in their apodictic necessity see Gutland (2021).
